# Crippling Side Effects Induced by Paliperidone Palmitate Treatment: A Case Report

**DOI:** 10.7759/cureus.13588

**Published:** 2021-02-27

**Authors:** Tamadhir Al-Mahrouqi, Ameera Al-Kindi, Ahmed Al-Harrasi

**Affiliations:** 1 Psychiatry, General Foundation Program, Oman Medical Specialty Board, Muscat, OMN; 2 Psychiatry, Psychiatry Residency Training Program, Oman Medical Specialty Board, Muscat, OMN; 3 Behavioural Medicine, Sultan Qaboos University Hospital, Muscat, OMN

**Keywords:** paliperidone palmitate, neuroleptic malignant syndrome, extrapyramidal side effects, dysphagia, tachycardia, urinary incontinence, blurred vision, hyperprolactinemia

## Abstract

In this report, we present the case of a 20-year-old woman with schizophrenia, who had been treated with a once-monthly dosage of long-acting paliperidone palmitate due to poor adherence to oral antipsychotics. She presented to the emergency department of the Sultan Qaboos University Hospital (SQUH), Muscat, Oman, with tachycardia, difficulty in breathing, difficulty in swallowing, choking, excessive production of saliva, drooling, urinary incontinence, blurry vision, a shuffling gait, slowness of movement, stooped posture, muscle rigidity, tremor, and hyperprolactinemia. The paliperidone palmitate injections were discontinued and the patient underwent a treatment course with procyclidine, and she subsequently achieved full recovery within seven days. It appears that even though the long-acting paliperidone palmitate prescription had improved her psychotic symptoms, it also induced several potentially life-threatening conditions. This case report highlights the diagnostic challenge represented by the overlapping features of the neuroleptic malignant syndrome (NMS) and extrapyramidal side effects (EPS).

## Introduction

Schizophrenia is a psychotic disorder characterized by delusions, hallucinations, disorganized speech, disorganized or catatonic behavior, and negative symptoms. With regard to the treatment for schizophrenia [1], atypical antipsychotics are the first-line option, and injectable, long-acting paliperidone palmitate is an atypical antipsychotic that has been proven to be an effective and tolerable treatment option in both acute and maintenance therapy of schizophrenia [2]. Long-acting, injectable antipsychotics can overcome the challenge of poor oral medication adherence in patients with schizophrenia, thereby reducing relapse and hospitalization rates, and ultimately reducing overall healthcare costs [3].

The most common adverse events associated with paliperidone palmitate treatment are injection site reactions, extrapyramidal symptoms, hyperprolactinemia, sedation, hypersalivation, orthostatic hypotension, tachycardia, and weight gain. The other, rarer side effects are neuroleptic malignant syndrome (NMS), tardive dyskinesia, and seizures [4].

In this report, we discuss the case of a schizophrenic patient presenting with shortness of breath, tachycardia, dysphagia, sialorrhea, urinary incontinence, hyperprolactinemia, blurry vision, and extrapyramidal side effects (EPS), due to an adverse drug reaction caused by the use of a once-monthly dose of long-acting paliperidone palmitate.

## Case presentation

A 20-year-old female student, who had been diagnosed with schizophrenia eight months previously, had initially presented with auditory hallucinations, persecutory delusions, and social withdrawal and self-neglect, which had led her to postpone two university semesters. Initially, treatment with oral olanzapine 5 mg once daily had been started. However, due to poor oral medication adherence, paliperidone palmitate injection had been prescribed to the patient as a once-monthly dose, with a loading dose of 150 mg on the first day, 100 mg on the eighth day, followed by a maintenance dose of 100 mg every four weeks. During a routine follow-up visit, the patient had shown a gradual improvement of her psychotic symptoms, and she had resumed attending university. However, she had gradually started to experience feelings of restlessness with the inability to sit still, blurry vision, and generalized slowness in movement. One week after her fifth maintenance dose of paliperidone (100 mg), she presented to the emergency department of the Sultan Qaboos University Hospital (SQUH), with breathing difficulties, intermittent chest pain, and palpitations while at rest. The patient reported difficulty in swallowing solid food, with episodes of choking and reduced oral intake. Moreover, she complained of excessive saliva production with drooling even when she was not eating.

The patient had also suffered from several episodes of involuntary leakage of urine. She experienced no difficulty in urinating, no urge or stress incontinence, and she had not experienced enuresis during childhood. She had also developed marked muscle stiffness, and required assistance with daily activities, such as showering, putting on clothes, and changing position from sitting to standing. Additionally, she had developed severe tremors in both of her arms, which meant that she could not eat without assistance.

Upon physical examination, her vital signs were abnormal: temperature of 37 °C during day time and not associated with her menstrual cycle, heart rate of 110 beats per minute, respiratory rate of 30 breaths per minute, blood pressure of 111/67 mmHg, and arterial blood oxygen saturation of 100% on room air. The patient was dyspneic and required accessory muscle use. She had a shuffling gait, extreme slowness of movement, and a stooped posture. She was noted to have rigidity in her neck, with a restricted range of head movement.

The neurological examination showed that she had fine tremors, increased muscle tone with marked rigidity, brisk deep tendon reflexes, and power of 4/5 in all four extremities. Examination of the respiratory and cardiovascular systems appeared unremarkable. 

Laboratory testing showed markedly elevated prolactin levels (six times the upper limit of normal) and a mild coagulation profile derangement, as listed in Table 1. An electrocardiogram revealed sinus tachycardia.

The patient was administered a normal saline infusion, 1 g of paracetamol intravenously, and procyclidine (10 mg) intramuscularly. Her scheduled paliperidone palmitate injections were discontinued.

**Table 1:** Patient’s laboratory test results

Variables	At presentation	At seven-day follow-up	Normal range
Hemoglobin (g/dl)	13.7	12.2	11-14.5
Platelet count (10^9^ per liter)	303	242	150-450
White blood cell count (10^9^ per liter)	4.6	5.5	2.4-9.5
Neutrophils (10^9^ per liter)	3.4	4.1	1-4.8
Lymphocytes (10^9^ per liter)	0.8	0.9	1.2-3.8
Creatinine kinase (U/l)	165	213	26-192
Serum prolactin (mIU/l)	2,927	-	102-496
Prothrombin time (seconds)	11.6	10.5	10.5-12.7
Activated partial thromboplastin time (seconds)	22.9	25	24.1-35.1
International normalized ratio	1.01	0.91	0.92-1.08
Sodium (mmol/l)	138	133	135-145
Potassium (mmol/l)	4.2	4.3	3.5-5.1
Estimated Glomerular filtration rate (ml/min/1.73 m^2^)	82	>90	
Creatinine (umol/l)	77	57	45-84
Free thyroxine (pmol/l)	22.4	18.7	12.3-20.2
Thyroid-stimulating hormone (pmol/l)	2.08	2.12	0.51-4.30
Magnesium (mmol/l)	0.99	0.91	0.70-0.91
Alanine aminotransferase (U/l)	14	36	0-33
Aspartate aminotransferase (U/l)	24	31	0-32
Alkaline phosphatase (U/l)	63	54	35-104
Albumin adjusted calcium (mmol/l)	2.22	-	2.15-2.55
Phosphate (mmol/l)	1.02	-	0.81-1.45

Hospital admission and supportive care were advised. However, the patient, who exhibited an intact sense of judgment and insight, refused to be admitted; she requested to be managed in the emergency department instead and was discharged as 'leave against medical advice,' with a prescription of oral procyclidine 5 mg three times a day. She attended a follow-up appointment seven days later and showed very rapid clinical improvement. She no longer suffered from dysphagia, sialorrhea, drooling, urinary incontinence, and EPS. However, she still had fine tremors in her hands. She was prescribed oral procyclidine 5 mg three times daily for two weeks and then two times daily for the following two weeks. At the next follow-up visit, the patient reported that she was not suffering from any psychotic symptoms. To maintain the remission of her symptoms, the patient was prescribed oral olanzapine 5 mg once daily, and she was found to be in good health during the following outpatient visits.

## Discussion

In this case report, we discussed a patient with schizophrenia who developed several potentially life-threatening conditions after receiving six injections of long-acting paliperidone palmitate. The differential diagnosis of drug-induced movement disorders includes NMS, akathisia, tremor, parkinsonism, and acute dystonic reactions [5]. NMS is a potentially life-threatening reaction to both typical and atypical antipsychotics. Our patient met the diagnostic criteria for NMS according to the fifth edition of the Diagnostic and Statistical Manual of Mental Disorders (DSM-5). The patient suffered from severe muscle rigidity associated with the use of neuroleptic medication as well as dysphagia, tremor, incontinence, and tachycardia [1]. Other classical signs are elevated temperature, altered consciousness, myoclonus, and autonomic instability, which were not detected in our patient. Supportive laboratory results for NMS include elevated creatine phosphokinase, myoglobinuria, leukocytosis, metabolic acidosis, hypoxia, elevated serum catecholamines, and low serum iron level. EPS may be challenging to diagnose and distinguish from NMS; the overlapping features between NMS and EPS are generalized rigidity with tremors, dyskinesia, and dysarthria [5].

The mechanism of action of paliperidone, like all other atypical agents, is through blocking dopamine 2 receptors, reducing positive symptoms of psychosis, and stabilizing affective symptoms. It also blocks serotonin 2A receptors, thereby inducing dopamine release in the frontal cortex, and as a consequence, it improves the cognitive functions and mood symptoms in patients with schizophrenia. EPS (such as acute dystonia, parkinsonism, and akathisia) and hyperprolactinemia are mediated by the antagonism of dopamine 2 receptors in nigrostriatal and tuberoinfundibular dopamine pathways, respectively [4].

First-generation antipsychotics are associated with a high rate of EPS, hyperprolactinemia, and cardiotoxicity compared to the newer atypical antipsychotics. On the other hand, although atypical antipsychotics are less likely to be associated with EPS, they are more likely to cause weight gain, metabolic syndrome, sexual dysfunction, sedation, and agranulocytosis. Most atypical antipsychotics are considered to be second-generation antipsychotics except for aripiprazole, which is referred to as third-generation due to its dopamine agonist effect at lower doses and antagonism effect in higher doses. Therefore, a low dose of aripiprazole could be effective in reducing hyperprolactinemia [6,7]. EPS may be alleviated by switching patients from typical antipsychotics to olanzapine or quetiapine. Moreover, quetiapine has been found to significantly reduce the severity of tardive dyskinesia [8].

Our patient experienced akathisia, manifested as the uncontrollable urge to move and a sense of restlessness, along with parkinsonian features such as generalized rigidity, bradykinesia, shuffling gait, hand tremors, and stooped posture. To the best of our knowledge, only two other cases have reported dysphagia following the administration of long-acting injectable paliperidone; both patients were young and diagnosed with schizophrenia, and one of the patients was managed successfully with promethazine [9], while the other patient received speech therapy, sensory input-triggered muscle relaxation, swallowing exercises, and articulation training [10]. It is vital to recognize the symptoms of dysphagia in patients taking antipsychotic medication, which would enable early intervention and the prevention of complications such as aspiration pneumonia, airway obstruction, and sudden death [11]. Our patient suffered from shortness of breath, dysphagia, and choking, which appeared to be related to acute dystonia. Laryngeal and pharyngeal dystonic reactions should always be assessed and may require emergency airway interventions.

Paliperidone-induced sialorrhea is a rare side effect. A meta-analysis by Harrington and English noted that only three patients out of 3,779 suffered from hypersalivation due to paliperidone use [12]. Sialorrhea has been found to occur secondary to existing dysphagia, as difficulty in swallowing causes salivary accumulation and ultimately drooling [13]. In another case of schizophrenia treated with paliperidone, it was reported that the patient suffered from sialorrhea without difficulty in swallowing and was managed successfully with the tricyclic antidepressant amitriptyline [14].

Another atypical finding in our case was that of isolated sinus tachycardia with no prior cardiovascular disease. Paliperidone palmitate can cause autonomic instability, which can include tachycardia and orthostatic hypotension. Furthermore, the existing evidence indicates that paliperidone palmitate extended-release tablets cause tachycardia in 3-18% of patients. The tachycardia has been found to improve gradually, with complete clearance of paliperidone palmitate metabolites, as was the case with this patient [15].

Intramuscular injections of paliperidone palmitate are rarely linked to urinary incontinence. One previous case report involved a patient diagnosed with schizophrenia who experienced urinary incontinence secondary to treatment with intramuscular depot paliperidone palmitate. The patient had a full recovery following the administration of solifenacin succinate [16].

Risperidone and paliperidone palmitate, second-generation antipsychotics, have been linked with elevated serum prolactin levels [17]. In addition, women of reproductive age have been found to exhibit a greater risk of developing hyperprolactinemia [18]. It is recommended to measure the fasting baseline prolactin levels before initiating treatment with antipsychotics and to re-measure the serum prolactin levels three months after treatment, and earlier if the patient becomes symptomatic [17]. Regular monitoring of serum prolactin levels in patients receiving antipsychotics helps avoid the direct and indirect consequences of hyperprolactinemia, such as galactorrhea, menstrual flow disturbances, ovulation disorders, and sexual dysfunction [19].

Management of antipsychotic-induced adverse effects includes lowering the dosage, stopping the suspected drug, switching to an antipsychotic with a different adverse-effect profile, and initiating concomitant medications if required. Lowering the drug dose to the lowest effective dose is relevant when the antipsychotic has proven to be beneficial in controlling psychotic symptoms, and the side effects are not medically serious. This is recommended especially with dose-related side effects, such as parkinsonism, sedation, hyperprolactinemia, and anticholinergic side effects. It is recommended to gradually switch to another antipsychotic if dosage adjustment does not reduce the emerging drug adverse effects. Treatment with a concomitant medication is usually the last resort. Anticholinergic medications are used to treat parkinsonism and acute dystonia, beta-adrenergic antagonists are used for akathisia, and topical atropine for sialorrhea [2]. For our patient, the paliperidone palmitate injection was discontinued and she was prescribed a treatment course with procyclidine, which improved the antipsychotic drug-induced adverse effects. The patient showed dramatic improvement in symptomology, indicating an effective treatment plan.

## Conclusions

Paliperidone palmitate has been reported to have good drug tolerance and efficacy. However, its long-term use may cause a number of potentially life-threatening side effects. Therefore, during the course of treatment with paliperidone palmitate, a thorough physical examination and careful monitoring at each follow-up visit is crucial for the early detection and management of emerging side effects. Although there is no consensus regarding the monitoring of the patient’s prolactin levels, measuring baseline levels should be considered prior to commencing treatment with antipsychotics.
